# A Novel Hybrid Approach for Partial Discharge Signal Detection Based on Complete Ensemble Empirical Mode Decomposition with Adaptive Noise and Approximate Entropy

**DOI:** 10.3390/e22091039

**Published:** 2020-09-17

**Authors:** Haikun Shang, Yucai Li, Junyan Xu, Bing Qi, Jinliang Yin

**Affiliations:** 1 Key Laboratory of Modern Power System Simulation and Control and Renewable Energy Technology, Ministry of Education, Northeast Electric Power University, Jilin 132012, China; 2201900107@neepu.edu.cn (Y.L.); 2201900164@neepu.edu.cn (J.X.); qibing@neepu.edu.cn (B.Q.); 2 School of Electrical and Electronic Engineering, Tianjin University of Technology, Tianjin 300384, China; searchforever@tjut.edu.cn

**Keywords:** partial discharge, de-noising, complete ensemble empirical mode decomposition, approximate entropy, correlation coefficient analysis

## Abstract

To eliminate the influence of white noise in partial discharge (PD) detection, we propose a novel method based on complete ensemble empirical mode decomposition with adaptive noise (CEEMDAN) and approximate entropy (ApEn). By introducing adaptive noise into the decomposition process, CEEMDAN can effectively separate the original signal into different intrinsic mode functions (IMFs) with distinctive frequency scales. Afterward, the approximate entropy value of each IMF is calculated to eliminate noisy IMFs. Then, correlation coefficient analysis is employed to select useful IMFs that represent dominant PD features. Finally, real IMFs are extracted for PD signal reconstruction. On the basis of EEMD, CEEMDAN can further improve reconstruction accuracy and reduce iteration numbers to solve mode mixing problems. The results on both simulated and on-site PD signals show that the proposed method can be effectively employed for noise suppression and successfully extract PD pulses. The fusion algorithm combines the CEEMDAN algorithm and the ApEn algorithm with their respective advantages and has a better de-noising effect than EMD and EEMD.

## 1. Introduction

Insulation deterioration is one of the most critical faults in the power system. Partial discharge (PD) is an essential symptom of insulation deterioration. Effective PD detection plays an irreplaceable role in the evaluation of insulation conditions [1,2]. The electrical equipment’s operating environment is very complex, often surrounded by various kinds of noise. PD signal is very weak, and on-site interference will cause significant difficulties in signal detection and extraction [3]. To extract real and reliable PD signals, some necessary de-noising methods are needed in the on-site environment [4].

Recently, various kinds of de-noising methods have been applied in PD signal extraction. Wavelet transform is suitable for processing non-stationary signal with better time-frequency resolution performance [5,6,7]. It has been widely researched in PD signal de-noising and achieved excellent application effectiveness [8,9,10]. Dai et al. [8] present a denoising method based on improved portogram and wavelet transform for UHF PD signals. Simulation and practical tests show that this novel method can effectively suppress the periodic narrowband noise and random white noise in PD signals and has better performance than the traditional wavelet method. Ghorat et al. [9] propose a novel denoising algorithm for PD signals based on adaptive dual-tree complex wavelet transform. Simulation and experimental tests show that this new algorithm has superior performance in PD noise reduction in comparison with other methods. Sun et al. [10] employ a new signal processing method based on wavelet packet to detect random PD signals in power cables. The wavelet packet threshold method was used to filter out residual white noise in the reconstructed PD signal. The encouraging performance demonstrates the effectiveness of the proposed algorithm. However, owing to the manual selection of mother wavelets and decomposition levels in the above studies, the wavelet method has some inherent limitations in practical applications. Empirical mode decomposition (EMD) provides a fully automatic decomposition technique for non-stationary and nonlinear signals [11,12,13]. EMD decomposes the original signal into several different intrinsic mode functions (IMFs). EMD does not need a predefined basis function and decomposition level. It has been widely applied in PD signal analysis and achieved a good effect [14,15,16]. Chen et al. [14] propose a data compression denoising method based on EMD to extract real PD signals under large disturbances. Simulation and actual signal results demonstrate that the proposed algorithm does not lose the original signal energy and can achieve better performance than other methods. Zhang et al. [15] employ EMD for UHF PD signal denoising. The results show that, compared with the conventional wavelet method, this method achieves a higher signal-to-noise ratio and slighter waveform distortion. Wang et al. [16] present a PD diagnostic method based on neural network and EMD. The actual measurements demonstrate that the proposed method is applicable to power capacitor PD detection. However, EMD has an inherent limitation of mode mixing problems that limits its wider use. To deal with the above issues, ensemble empirical mode decomposition (EEMD) was proposed by Huang based on EMD [17,18,19]. White noise components are added manually in EEMD and eliminated through repetitive averaging. EEMD could effectively overcome the mode mixing in EMD by decomposing the original noise-corrupted signals into IMFs. Jin et al. [20] present a novel adaptive EEMD method for switchgear PD signal denoising. The simulation and experimental results show the effectiveness and superiority of the proposed method compared with the conventional wavelet algorithm and EMD-based method. Chan et al. [21] propose a self-adaptive technique for PD signal denoising with automatic threshold determination based on EEMD and mathematical morphology. The simulated and real PD results show that the proposed denoising algorithm is superior to wavelet transform and EMD-based PD de-noising methods. However, in the above research, the white noise added to the decomposing process of EEMD is not easily eliminated, which will cause reconstruction errors.

To improve the de-noising ability of EEMD, complete ensemble empirical mode decomposition with adaptive noise (CEEMDAN) was introduced [22,23,24,25]. In CEEMDAN, adaptive noise is added into the signal decomposition. The residual noise in IMFs can be reduced by changing the decomposition process. This method can overcome the shortcomings of EEMD as well as improve the operational efficiency and reconstruction accuracy. Approximate entropy (ApEn) is a non-negative number that can measure the complexity of time series [26,27]. It represents the complexity degree in different scales and frequencies of signals. It is suitable for non-stationary signal analysis. The more complex the time series, the larger the ApEn value. ApEn has been studied in nonlinear analysis of various kinds of complex signals and achieved a good effect [28,29,30].

The proposed signal de-noising technique combines the advantages of CEEMDAN and ApEn. In this paper, CEEMDAN is utilized for PD signal decomposition to eliminate the mode mixing phenomenon in conventional EMD and improve the reconstruction accuracy in EEMD. ApEn of each IMF is then calculated to measure the complexity of PD signals. To select useful IMFs representing prominent PD features, correlation coefficient analysis (CCA) between IMFs and original signals is introduced [31]. Real IMFs can then be extracted according to ApEn and CCA for PD signal reconstruction. To verify the de-noising effect and superiority of the proposed method, EMD and EEMD are introduced into this paper. The simulation and on-site PD results demonstrate its effectiveness and superiority.

The rest of this paper is organized as follows. Section 2 and Section 3 introduce the conception of CEEMDAN and approximate entropy. Section 4 presents the PD de-noising approach based on CEEMDAN-ApEn. Section 5 describes PD signal simulation and gives de-noising results with simulated signals. Section 6 evaluates the proposed method’s performance with experimental and on-site signals and compares it with different de-noising methods. Section 7 concludes this paper.

## 2. Review of CEEMDAN

EEMD represents an extension of EMD. During EEMD decomposition, the noise added into the original signals cannot be eliminated, which may cause reconstruction errors [32]. On the basis of EEMD, CEEMDAN was proposed by Colominas et al. for further elimination of the mode mixing phenomenon. It is suitable for non-stationary signals thanks to its small iteration numbers and high convergence performance.

In CEEMDAN decomposition, adaptive white noise is introduced into each residual component [33]. The algorithm procedure will briefly be discussed in the following steps.

Step 1: Add a noise $w^{i}(t)$ to the original signal x(t):

(1) $$X^{i}(t)=x(t)+w^{i}(t)$$

in which $w^{i}(t)(i=1,2,\ldots ,N)$ meets the Gauss distribution and *N* is the number of samples.

Step 2: Decompose $X^{i}(t)$ using EMD into IMF $F_{1}^{i}$. By averaging $F_{1}^{i}$, the first component of IMF can be obtained in Formula (2).

(2) $${\tilde{F}}_{1}(t)=\frac{1}{N}\sum_{i=1}^{N}F_{{}_{1}}^{i}$$

Step 3: Calculate the first residual component $r_{1}(t)$.

(3) $$r_{1}(t)=x(t)-{\tilde{F}}_{1}(t)$$

Step 4: Calculate the second IMF.

(4) $${\tilde{F}}_{2}(t)=\frac{1}{N}\sum_{i=1}^{N}E_{1}(r_{1}(t)+\varepsilon_{1}E_{1}[w^{i}(t)])$$

where $E_{j}(\cdot )$ represents the *j*th IMF of the signal, and $\varepsilon_{j}$ is the parameter of white noise power.

Step 5: Calculate the *k*th residual component as follows:

(5) $$r_{k}(t)=r_{k-1}(t)-{\tilde{F}}_{k}(t)$$

where k=2,3,…,K and *K* represents the highest order of IMF.

Step 6: Calculate the (*k +* 1)^th^ IMF component as follows:

(6) $${\tilde{F}}_{k+1}(t)=\frac{1}{N}\sum_{i=1}^{N}E_{1}(r_{k}(t)+\varepsilon_{k}E_{k}[w^{i}(t)])$$

Step 7: Repeat Stage (5) and (6) until the residual component cannot be subdivided. The final residual component can be described as follows:

(7) $$R(t)=x(t)-\sum_{k=1}^{K}{\tilde{F}}_{k}(t)$$

Step 8: The final signal can be decomposed as follows [22]:

(8) $$x(t)=\sum_{k=1}^{K}{\tilde{F}}_{k}(t)+R(t)$$

## 3. Review of Approximate Entropy

Approximate entropy (ApEn) is a non-negative number used for the complexity measurement of time series [34]. It is widely used in the analysis of nonlinear characteristics of complex signals. The calculation of ApEn can be realized through the following steps.

Suppose the original time series u(i),i=0,1,…,N, in which *N* is the number of data sets.

Step 1: The time series {*u*(*i*)} can be extended to *m*^th^ vector *X*(*i*), which can be defined as follows:

(9) X(i)=[u(i),u(i+1),…,u(i+m−1)]

where x=1,2,…,N−m+1 and *m* is the pattern dimension.

Step 2: Calculate the distance between *X*(*i*) and *X*(*j*).

(10) $$d[X(i),X(j)]=\underset{k=0,1,\ldots ,m-1}{\max}\left|u(i+k)-u(j+k)\right|$$

where j=1,2,…,N−m+1.

Step 3: Calculate the ratio of *n*(*d*) to *n*(*t*), which is defined as $C_{i}^{m}(r)$.

(11) $$C_{i}^{m}(r)=\frac{n(d)}{n(t)}$$

where *n*(*d*) means the number of *d*[*X*(*i*), *X*(*j*)] < *r*, *n*(*t*) = *N* − *m* + 1 is the total number of vectors, and *r* > 0 is the preset threshold.

Step 4: Calculate the logarithm to $C_{i}^{m}(r)$ and the mean value can be obtained as follows:

(12) $$\Phi^{m}(r)=\frac{1}{N-m+1}\sum_{i=1}^{N-m+1}\ln C_{i}^{m}(r)$$

Step 5: The ApEn can be defined as follows [26]:

(13) $$ApEn(m,r,N)=\Phi^{m}(r)-\Phi^{m+1}(r)$$

in which $\Phi^{m+1}(r)$ can be obtained through Steps (1)–(4).

## 4. PD Signal De-Noising Based on CEEMDAN and ApEn

### 4.1. Algorithm Principle

Aimed at the non-stationary and non-linearity of PD signals, CEEMDAN is employed to analyze original signals. Compared with traditional EEMD, this approach has a higher extraordinary ability to discriminate different frequency parts. Firstly, CEEMDAN decomposes noisy PD signals into IMFs, which contain both noise parts and real signals. Secondly, the ApEn values of each IMF are calculated. These values can describe the irregularity and complexity of PD signals. The higher the complexity, the larger the value. According to the principle of ApEn, those IMFs that represent dominant signal features can be selected. Then, the correlation coefficient (CC) between each IMF and the original PD signal is calculated. CC values can effectively measure the similarity degree between two signals. After that, a threshold is set to eliminate those IMFs with low similarity. Finally, real IMFs are obtained for PD signal reconstruction.

### 4.2. Algorithm Procedure

1. Extract the original PD signals. Because of the complexity of power transformers’ field environments, PD signals are always interfered with by various noises.
2. Decompose the original PD signal into different IMFs with CEEMDAN. These IMFs may consist of real PD information and complex noise interference.
3. Calculate ApEn values of IMFs extracted from CEEMDAN decomposition. ApEn represents the complexity of signals in different scales and frequencies. Owing to the non-stationary of original PD signals, the ApEn values may be different from each other.
4. Remove those IMFs that represent noise components. According to ApEn theory, noise and PD signals can be distinguished by different ApEn values. If the ApEn value is above a certain threshold, then the IMF is regarded as noise and abandoned. Otherwise, the IMF contains PD information and will be kept.
5. Calculate the correlation coefficient between the original PD signal and each selected IMF. As known, the CC value of the IMF that contains little PD information will be small. Therefore, real IMFs similar to the original signal will be selected as final parts through a certain threshold.
6. Reconstruct clean PD signal with final IMFs. These IMFs contain dominant PD features and show a strong correlation with original signals. This reconstruction method can efficiently recover the clean PD pulses from noisy signals.

**Figure 1:**
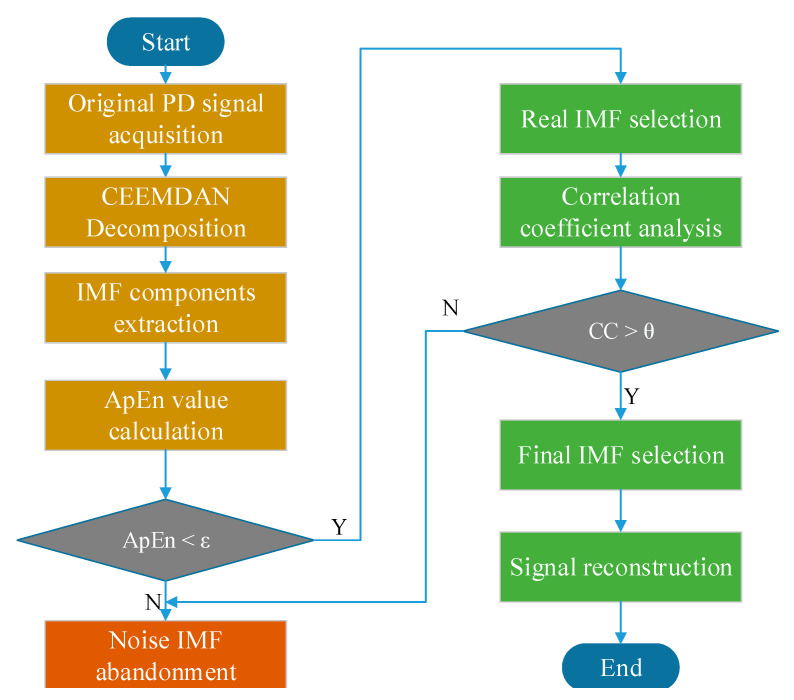
De-noising procedure based on complete ensemble empirical mode decomposition with adaptive noise (CEEMDAN) and approximate entropy (ApEn). PD, partial discharge; IMF, intrinsic mode function; CC, correlation coefficient.

The main de-noising procedure based on CEEMDAN and ApEn is shown in Figure 1.

## 5. Simulation Analysis

### 5.1. Simulated Signal

A substantial amount of studies indicate that partial discharge is a non-stationary and high-frequency signal. High-frequency PD pulses can be expressed by mathematical models as follows [35]:

(14) $$s_{1}(t)=Ae^{-t/\tau }\sin2\pi f_{c}t$$

(15) $$s_{2}(t)=A(e^{-1.3t/\tau }-e^{-2.2t/\tau })\sin2\pi f_{c}t$$

where *A* is the signal amplitude, τ is the attenuation coefficient, and $f_{c}$ is the oscillation frequency.

**Figure 2:**
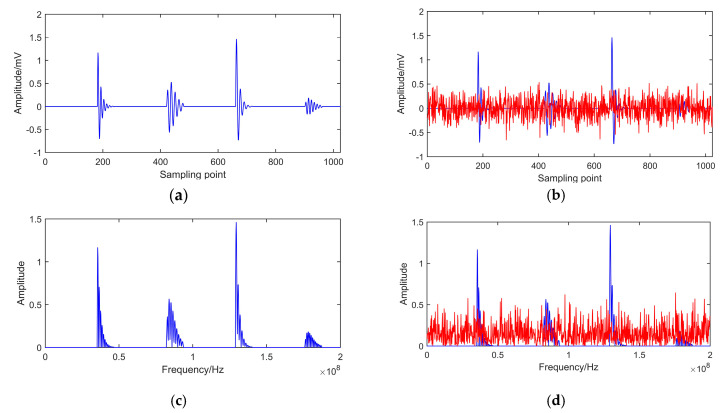
Simulated PD signal. (**a**) Original PD signal. (**b**) Noisy PD signal. (**c**) Spectrogram of original signal. (**d**) Spectrogram of noisy signal.

The sampling frequency is 150 MHz and the number of samples is 1024. The sampling parameters of four different PD pulses are shown in Table 1. The simulated signal is shown in Figure 2a.

Owing to the poor operational environment of electrical equipment, the on-line detection of PD signals is usually influenced by environmental inference, mostly the white noise. The white noise is added to simulate the real PD signal, which satisfies Gauss distribution $N(0,0.02^{2})$. A simulated noisy signal is shown in Figure 2b. The spectrograms of signals are shown in Figure 2c–d.

From Figure 2, it can be seen that the added noise considerably corrupts clean PD pulses. Four pulses are completely immersed in random noise. The clean signal cannot be recognized for the second and fourth pulse because of the small amplitude. To extract effective real PD signals, we consider in this paper a few specific de-noising methods.

### 5.2. Signal Decomposition

**Figure 3:**
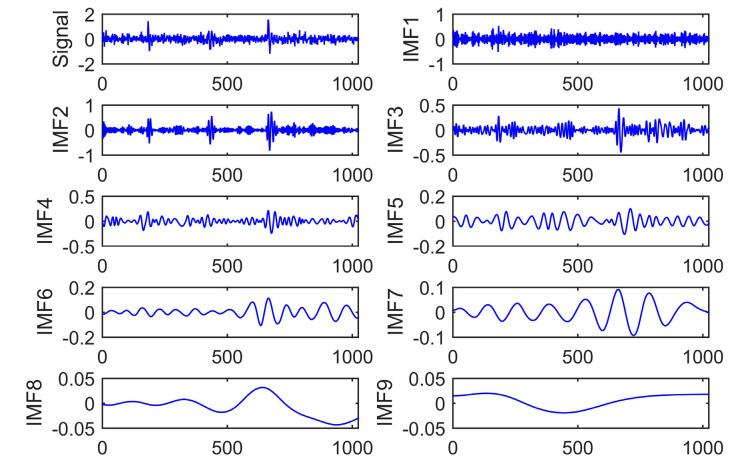
PD signal decomposition based on EMD.

**Figure 4:**
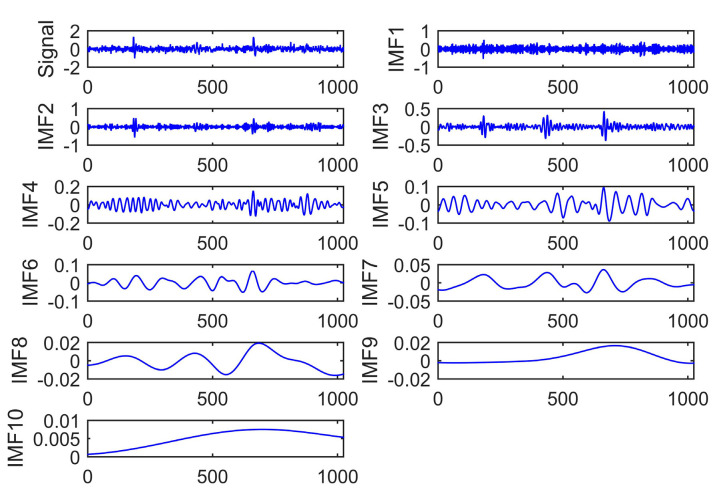
PD signal decomposition based on EEMD.

**Figure 5:**
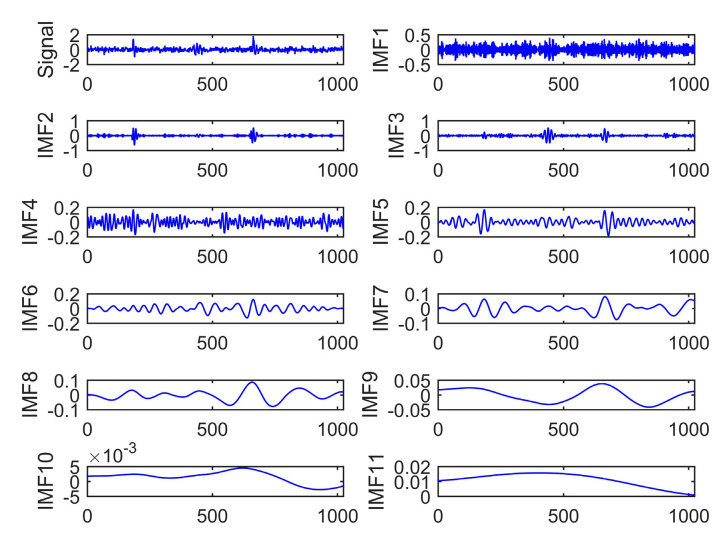
PD signal decomposition based on CEEMDAN.

To verify the proposed algorithm’s effectiveness and superiority, EMD and EEMD algorithms are employed to analyze the PD signal. Figure 3, Figure 4 and Figure 5 show the signal decomposition using different methods, including EMD, EEMD, and CEEMDAN. For EEMD and CEEMDAN, 100 groups of Gaussian white noise with standard deviation of 0.2 were added into the original signal.

It can be seen from Figure 3 that eight IMF components and a single residual component were obtained through EMD decomposition. IMF1 has the highest component frequency. The nature of signals cannot be analyzed accurately. Besides, the substantial similarity between IMF6 and IMF7 indicates that the mode mixing phenomenon exists in EMD decomposition.

The noisy PD signal decomposition result based on EEMD is presented in Figure 4. The standard deviation of white Gaussian noise is 0.2, and the repetitive number is 200. Figure 4 shows that the number of IMFs decomposed by EEMD is ten, which is greater than that of EMD. This means more details of signals can be found out through EEMD. The white noise makes each IMF maintain the continuity in the time domain. The EEMD decomposition method could obtain frequency components of the original PD signal. However, IMF 1–3 show that a particular oscillation phenomenon occurs during signal component analysis. This suggests that the white noise added in EEMD causes unfavorable influence on signal decomposition. Some necessary steps need to be taken to control noise’s impact to ensure the accuracy of detection.

Figure 5 presents the IMFs’ components decomposed by CEEMDAN. It is clear that ten IMF components and a single residual component were obtained. This decomposition method makes it more uniform in the distribution of the IMFs. Besides, the frequency changes between different IMF components have become more apparent. The detailed decomposition helps CEEMDAN solve the problems of mode mixing even further.

**Figure 6:**
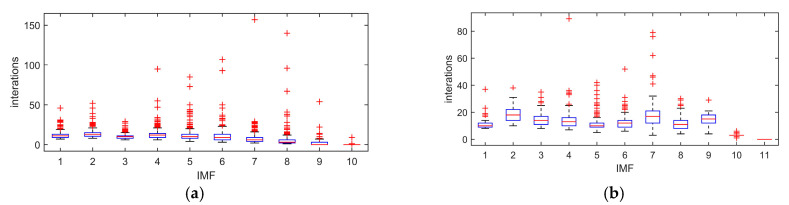
Iterations of each IMF. (**a**) EEMD. (**b**) CEEMDAN.

The boxplots of computation numbers of IMFs using different decomposition methods are shown in Figure 6. It shows that, for each IMF selection, the number of calculations with CEEMDAN is smaller than that of EEMD. This means the CEEMDAN method can reduce the operation time and improve the efficiency of signal decomposition.

### 5.3. Approximate Entropy Calculation

It is shown from Section 5.2 that eleven IMFs are obtained by CEEMDAN decomposition. However, the number of IMF components of PD signals may vary with different trials. Therefore, it is inaccurate to estimate the boundary between noise and PD signals by subjective judgment. To analyze the complexity of PD signals and reduce the reconstruction error, ApEn is introduced to distinguish between noise and real PD pulses. Before calculation, the pattern dimension *m* and similar tolerance boundary *r* should be predefined. On the basis of the researchers’ previous study experience [36], the parameters are defined as follows.

(16) $$\left\{ \begin{array}{l} m=2\\ r=0.2E_{SD} \end{array}\right.$$

where $E_{SD}$ is the standard deviation of original signals.

**Figure 7:**
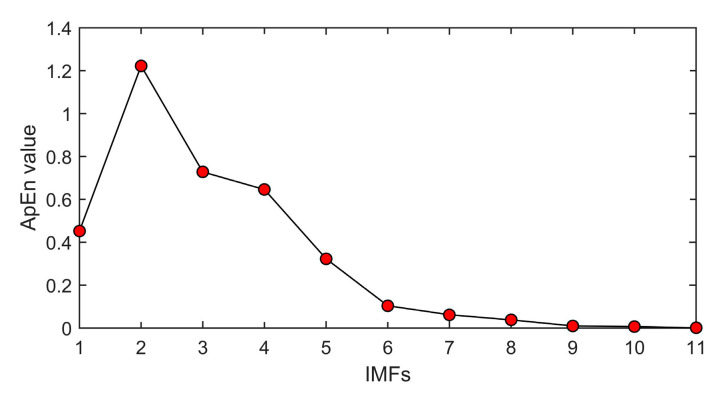
ApEn values of IMFs.

The value of approach entropy of each IMF is calculated. Through multiple calculations, the mean value of ApEn is shown in Figure 7.

Figure 7 shows that different IMFs possess different ApEn values, which means diverse complexity exists in the decomposition levels. It can be concluded that, from A2 to A11, the ApEn values decrease gradually. This illustrates that the complexity of each IMF is gradually reduced. According to the theory of ApEn, more irregular time series obtain greater ApEn values. Owing to the randomness and irregularity of white noise, ApEn values of noisy IMFs will be greater than those of clean ones. In this paper, a threshold ε is set to 0.5 to eliminate noise IMFs. If the ApEn value is greater than ε, the IMF will be abandoned as a noisy part. Otherwise, the IMF will be kept as a clean part. After a comparison of different ApEn values, IMF 1 and 5–11 are retained.

### 5.4. Correlation Coefficient Analysis

Through Section 5.3, those IMFs that represent noise parts are effectively eliminated. However, some over-decomposition phenomenon may occur during CEEMDAN, which can produce pseudo-component in IMFs. It will be even worse when the signal gets more complicated. This will also cause reconstruction errors. In this paper, to extract useful and effective IMF components, correlation coefficient analysis is employed for IMF selection. The CC is defined as follows [37].

(17) $$CC=\frac{\sum_{i=1}^{k}\left(x_{i}-\bar{x})(imf_{i}-\bar{imf}\right)}{\sqrt{\sum_{i=1}^{k}{\left(x_{i}-\bar{x}\right)}^{2}}\sqrt{\sum_{i=1}^{k}{\left(imf_{i}-\bar{imf}\right)}^{2}}}$$

where *x* is the original signal, $\bar{x}$ is the mean value of *x*, *imf* is the IMF component, $\bar{imf}$ is the mean value of IMF, and *k* is the number of IMF components.

Firstly, the CC value of each IMF is calculated using Formula (17). Eight CC values are obtained shown in Table 2. The CC value can effectively quantify the similarity between two different time series. Table 2 shows that different IMFs have a certain degree of similarity with the original PD signal.

To eliminate those IMFs that have low similarity with original PD signals, a threshold *θ* is preset. If the CC value is greater than *θ*, the IMF will be kept as an effective component. Otherwise, the IMF will be abandoned as a useless part. In this paper, the threshold is defined as follows.

(18) $$\theta =\sqrt{\frac{\sum_{i=1}^{k}{\left(CC_{i}-\bar{CC}\right)}^{2}}{k}}$$

After multiple trials, *θ* is set to 0.6. According to CC’s principle, IMF 1, IMF 9, IMF 10, and IMF 11 are removed as a result of CC’s small value. Those IMFs that have less similarity with original PD signals are abandoned. The remaining IMFs have a strong correlation with original signals, which means they possess prominent information on PD features.

### 5.5. De-Noising Results Analysis

To compare the performance of different de-noising methods, EMD, EEMD, and CEEMDAN-ApEn are employed to de-noise the simulated PD signal. Three evaluation indexes are used for quantitative analysis of the quality of signal de-noising, including the signal to noise ratio (SNR), mean square error (MSE), and normalized correlation coefficient (NCC). The higher the SNR and NCC, the more effective the de-noising result. The smaller the MSE, the more similar the original and the de-noised signal. SNR, MSE, and NCC are defined as follows:

(19) $$SNR=10\log\frac{\sum_{i=1}^{n}s^{2}(i)}{\sum_{i=1}^{n}{\left(s(i)-\hat{s}(i)\right)}^{2}}$$

(20) $$MSE=\frac{1}{n}\sum_{i=1}^{n}\left({\left|s(i)-\hat{s}(i)\right|}^{2}\right)$$

(21) $$NCC=\frac{\sum_{i=1}^{n}s\cdot \hat{s}}{\sqrt{\sum_{i=1}^{n}s^{2}\sum_{i=1}^{n}{\hat{s}}^{2}}}$$

where s(i) is the original PD signal and $\hat{s}(i)$ is the de-noised signal.

**Figure 8:**
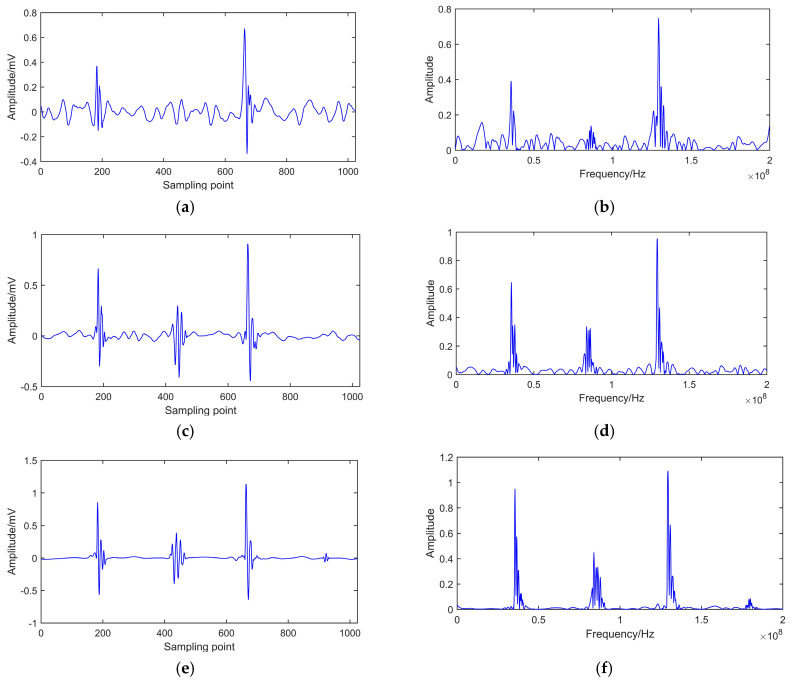
De-noised PD signal in time and frequency domain. (**a**) EMD de-noising. (**b**) Spectrum of EMD de-noising. (**c**) EEMD de-noising. (**d**) Spectrum of EEMD de-noising. (**e**) CEEMDAN-ApEn de-noising. (**f**) Spectrum of CEEMDAN-ApEn de-noising.

De-noising results with different methods are shown in Figure 8a–f.

Figure 8a,b shows that the fourth pulse, which has a smaller amplitude, cannot be detected after EMD de-noising. Furthermore, there is an apparent oscillating phenomenon after de-noising. Figure 8c,d indicates that the oscillating phenomenon can be restrained to a certain extent after EEMD. However, the fourth pulse still cannot be detected. It can be seen from Figure 8e,f that all of the PD pulses could be detected, and a smooth signal is achieved using the CEEMDAN-ApEn method.

It can be concluded from the above results that the oscillating phenomenon was produced as a result of mode mixing after EMD decomposition. The unidentified PD pulse was caused by considering detailed information in the IMF during the de-noising process. Part of critical information was lost after EMD. Adding the white Gaussian noise during EEMD could ensure the continuity of each IMF in the time domain. It can eliminate the mode mixing phenomenon to a certain extent. However, because of the influence of added white noise during EEMD decomposition, some oscillation distortion still cannot be suppressed entirely. Consequently, the fourth pulse still cannot be detected clearly, and some useful frequency spectral components are missing. In CEEMDAN-ApEn, adaptive white noise was added into each residual component, which leads to complete decomposition. It can solve the problem of mode mixing further. ApEn can measure the non-linearity and complexity of time series. ApEn values can easily recognize noisy parts in IMF. Combined with CEEMDAN and ApEn, useful IMFs can be selected for PD signal reconstruction. After CEEMDAN-ApEn, all PD pulses were successfully identified. Moreover, the de-noised signal was very smooth, and clear frequency components were also obtained.

SNR, MSE, and NCC results with different de-noising methods are shown in Table 3.

It can be seen from Table 3 that the SNR and NCC are the largest, and the MSE is the smallest using the CEEMDAN-ApEn method, which means the de-noising effect is the best. Compared with EMD and EEMD, CEEMDAN-ApEn can eliminate the mode mixing phenomenon in EMD and reduce the influence of added noise in EEMD decomposition. The de-noised signal could well represent the characteristics of the original PD signal.

**Figure 9:**
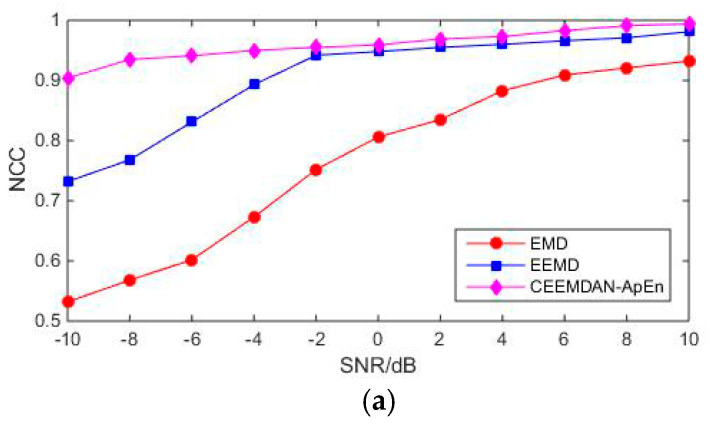

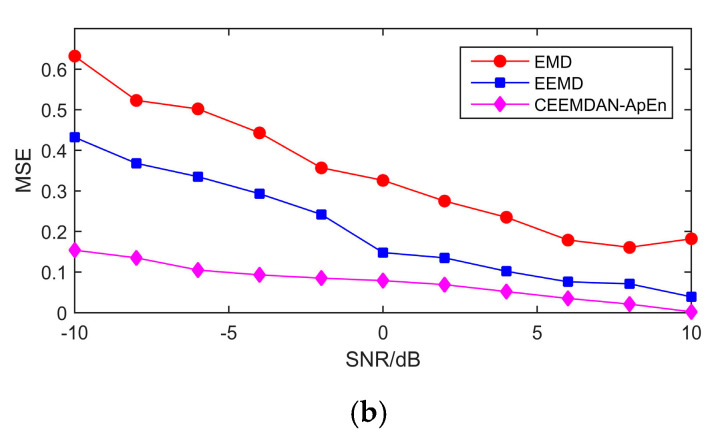
De-noised results of different methods. SNR, signal to noise ratio; MSE, mean square error; NCC, normalized correlation coefficient. (**a**) NCC vary with different SNR. (**b**) MSE vary with different SNR.

To verify the effectiveness of the de-noising method in different noise environments, PD simulation results under different SNR conditions are discussed in detail. Figure 9 presents the results of different de-noising algorithms varying with diverse SNR.

Figure 9 shows that the three algorithms’ de-noising performance is different from the increase of SNR. It can be seen from Figure 9a that the NCC value of CEEMDAN-ApEn is larger in each SNR than that of EMD and EEMD. This means that the proposed method has the highest similarity between the reconstructed and the original signal. Moreover, the performance of CEEMDAN-ApEn is relatively stable. Figure 9b illustrates that, compared with EMD and EEMD, CEEMDAN-ApEn obtained smaller MSE. It indicates that the de-noising effect of the proposed method is better than the other two traditional ones. It also shows the stable performance varying with SNR.

## 6. Experimental and On-Site PD Signal Analysis

### 6.1. Experimental PD Signal

**Figure 10:**
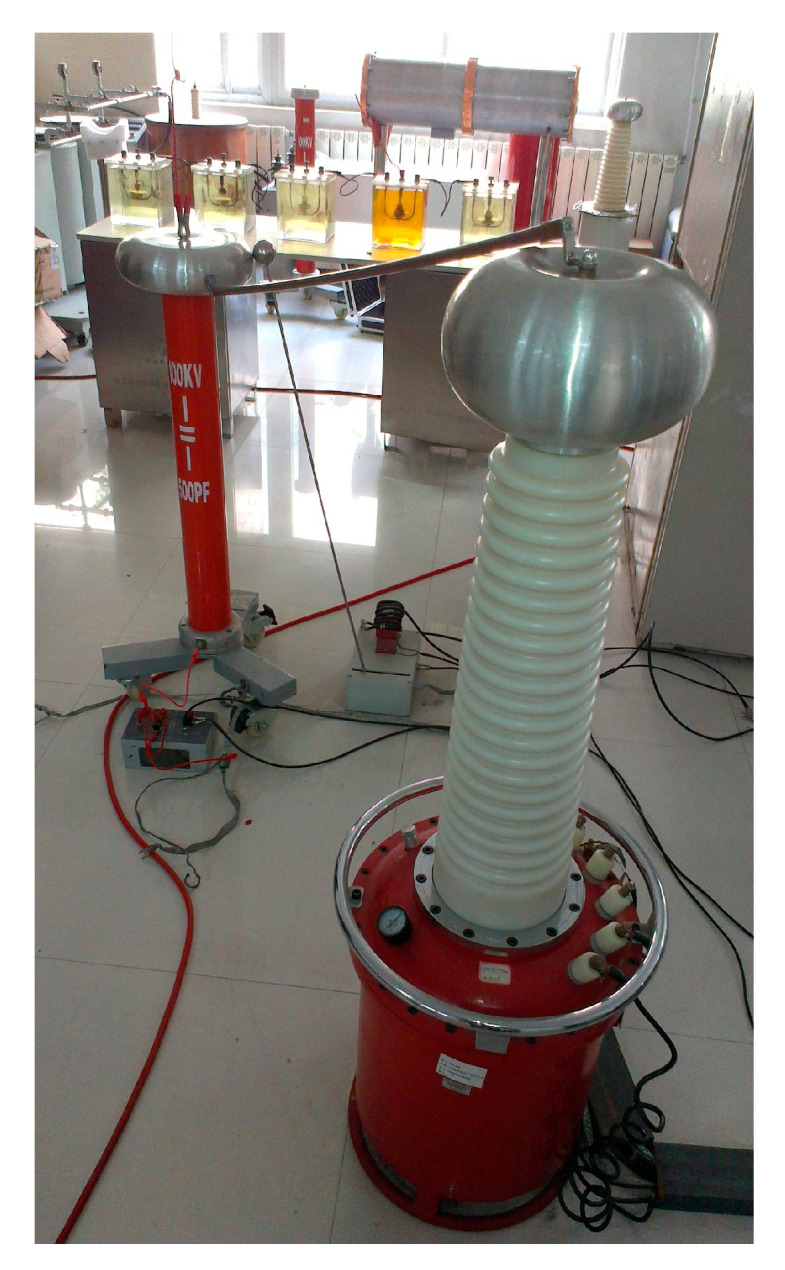
Experimental setup.

**Figure 11:**
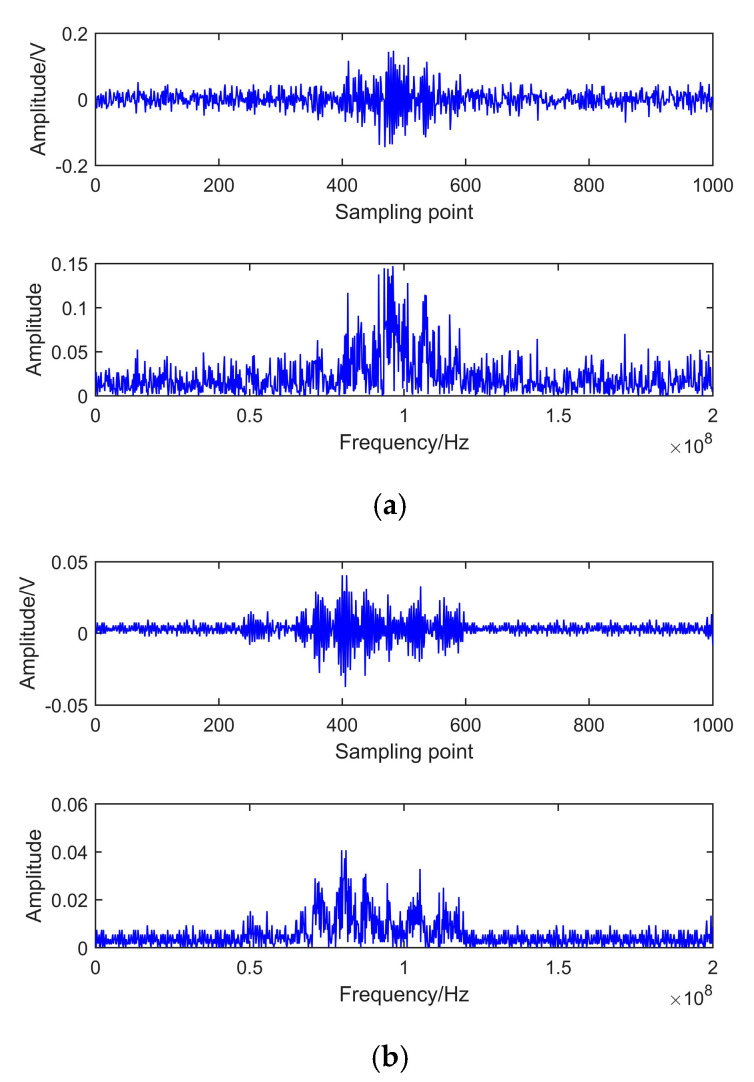
Measured PD signals in laboratory. (**a**) Measured signal 1. (**b**) Measured signal 2.

Figure 10 shows the circuit for PD simulation experiments. To verify the proposed de-noising method’s effectiveness, two PD signals are extracted under the noisy experimental environment. Measured signals are shown in Figure 11. It indicates that PD signals are disturbed by observed interference, and real PD pulses cannot be detected effectively. To remove the experimental interference mixed in PD pulses, certain denoising measures are taken as follows. Because of the randomness and unknown characteristics of noises, in this paper, the noise rejection ratio (NRR) is utilized to measure de-noising quality [38]. NRR is defined as follows:

(22) $$NRR=10(\mathrm{lg}\sigma_{1}^{2}-\mathrm{lg}\sigma_{2}^{2})$$

where $\sigma_{1}$, $\sigma_{2}$ represent the noise deviation of pre-treatment and post-treatment, respectively. The deviation can be defined as follows:

(23) $$\sigma =\sqrt{\frac{1}{Q}\sum_{d=1}^{Q}{\left(S_{d}-\mu \right)}^{2}}$$

where *Q* is the number of samples, *S_d_* represents the *d*th sampling signal, and μ is the mean of signal.

**Figure 12:**
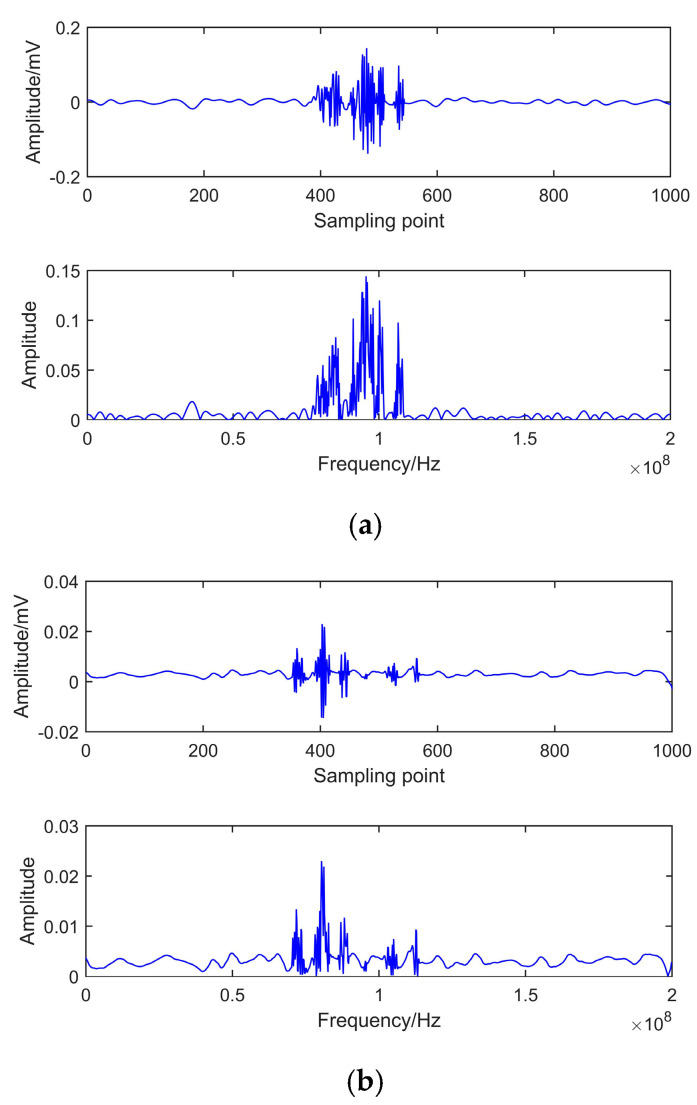
De-noising with EEMD. (**a**) Measured signal 1. (**b**) Measured signal 2.

**Figure 13:**
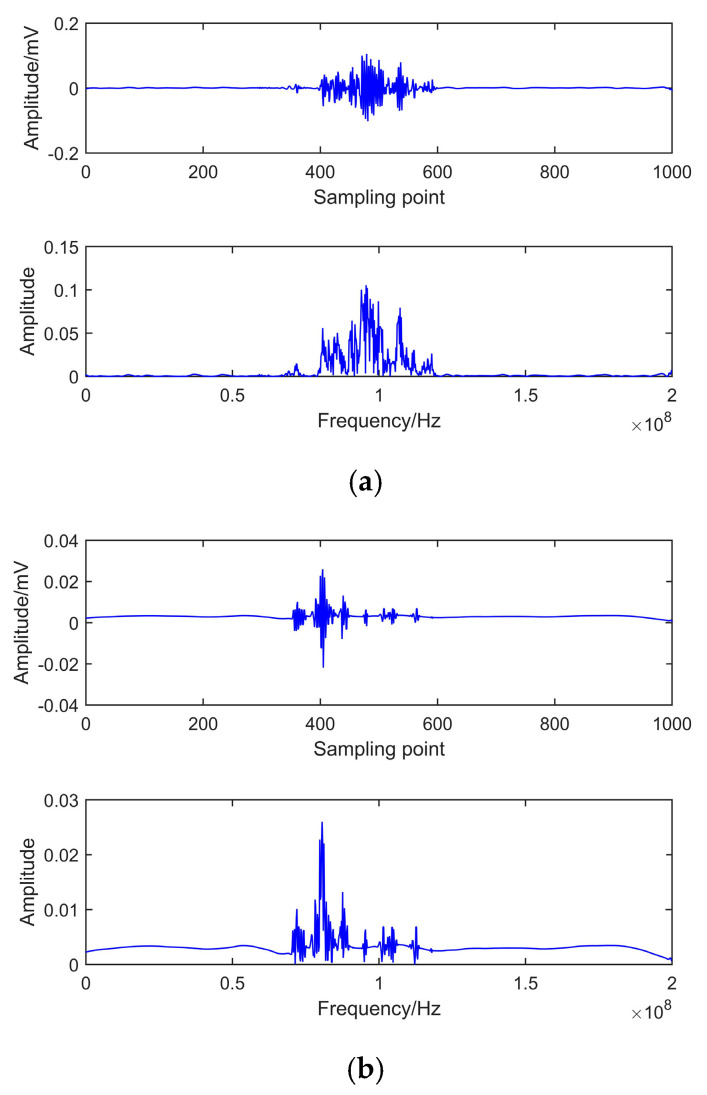
De-noising of CEEMDAN-ApEn. (**a**) Measured signal 1. (**b**) Measured signal 2.

NRR reflects the prominent level of de-noised signal. The signals de-noised by EEMD and CEEMDAN-ApEn are shown in Figure 12 and Figure 13. NRR results are shown in Table 4.

Figure 12 presents the time and frequency domain of de-noised results by EEMD. It shows that, after EEMD denoising, PD pulses could be detected. However, noticeable oscillation distortion exists in the de-noised signal. It can be seen from Figure 13 that all PD pulses were effectively extracted. Moreover, the oscillation almost disappeared after denoising. This means CEEMDAN-ApEn effectively reduces the added noise’s influence and improves the signal’s smoothness. Table 4 shows that a higher NRR is obtained after CEEMDAN-ApEn compared with EEMD. Above all, CEEMDAN-ApEn has an obvious advantage over EEMD on PD de-noising.

### 6.2. On-Site PD Signal

**Figure 14:**
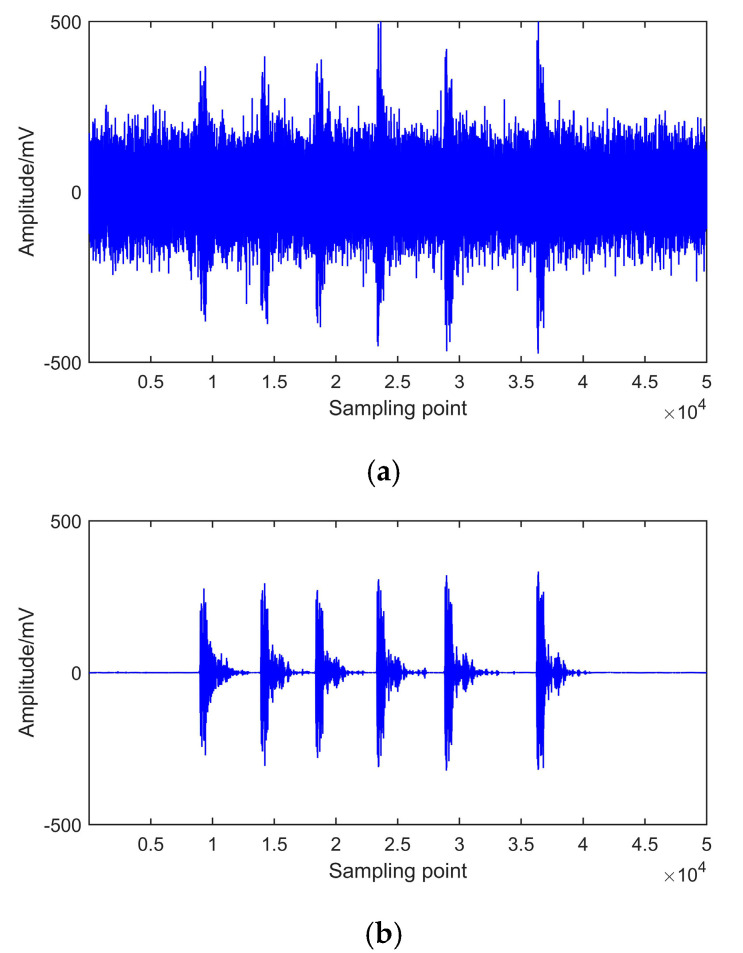
Analysis of on-site PD signal. (**a**) Field PD signal. (**b**) CEEMDAN-ApEn de-noising.

Figure 14a presents the on-site PD signal from the on-line monitoring system of one substation in Anhui Province. Because of the complex practical environment, PD signals are disturbed by environmental interference, and real PD signals cannot be detected effectively. To eliminate the environmental disturbance and extract effective PD pulses, CEEMDAN-ApEn is employed for signal analysis. The de-noised signal is shown in Figure 14b. It shows that PD pulses can be effectively extracted after de-noising using the proposed method.

## 7. Conclusions

Noise interference is a big problem in PD signal extraction. This paper presents an approach based on CEEMDAN-ApEn for PD de-noising. CEEMDAN can effectively separate the original signal into different intrinsic mode functions. ApEn of each IMF is calculated to measure the complexity of the signal. Those IMFs that contain noise components can be removed by ApEn analysis. Correlation coefficient analysis is then employed between IMFs and the original PD signal. Finally, effective IMFs are obtained for signal reconstruction by analyzing the correlation coefficient.

To verify the proposed approach’s effectiveness and superiority, EMD and EEMD are applied for PD signal analysis in this paper. EMD can decompose signals into several IMFs and restrain the noise to a certain extent. However, mode mixing and oscillation distortion restricted its application. EEMD can eliminate the mode mixing phenomenon by adding manual white noise into signal decomposition. However, the added noise cannot be easily eliminated, and the residual noise still exists, which may cause signal reconstruction errors. In CEEMDAN, adaptive white noise is added into signal decomposition, and its unique decomposition procedure realizes residual noise isolation. ApEn can measure the complexity and non-stationarity of the PD signal. Using the ApEn analysis of decomposed IMFs, those noisy components can be effectively removed from PD signals. Useful components that represent prominent PD features can be further selected for signal reconstruction according to the correlation coefficient analysis.

Simulated and on-site PD signals indicate that CEEMDAN can successfully eliminate the mode mixing problem and improve the reconstruction efficiency. The ApEn extracted from IMFs can represent the prominent components of PD features. Combining the advantages of CEEMDAN and ApEn, the proposed de-noising method can effectively suppress the inference in PD detection. Compared with EMD and EEMD, CEEMDAN-ApEn has a better de-noising effect through different denoising index analysis. It can effectively extract the weak PD pulse from strong background noise. In brief, the proposed approach has a good signal denoising effect and provides a new practical tool for PD signal detection.

It is noteworthy that the operation environment of field equipment is quite complex. PD signals extracted from sensors are usually contaminated by various kinds of noise, such as white noise, periodic narrowband noise, impulse noise, and so on. Moreover, different types of noise may cause various difficulties in real PD extraction. For further consideration, different types of noise could be added into PD simulation to verify the effectiveness of the proposed algorithm. Further, more complex on-site PD signals could be collected for deeper analysis.

## Figures and Tables

**Table 1:** Simulation parameters.

	Amplitude/mV	Attenuation Coefficient/us	Oscillation Frequency/MHz
Pulse 1	1.5	0.05	20
Pulse 2	3	0.1	15
Pulse 3	2	0.05	15
Pulse 4	1	0.1	20

**Table 2:** Correlation coefficient (CC) values. IMF, IMF, intrinsic mode function.

IMF1	IMF5	IMF6	IMF7	IMF8	IMF9	IMF10	IMF11
0.552	0.823	0.906	0.932	0.773	0.521	0.328	0.195

**Table 3:** De-noising index comparison. CEEMDAN, complete ensemble empirical mode decomposition with adaptive noise; ApEn, approximate entropy; SNR, signal to noise ratio; MSE, mean square error; NCC, normalized correlation coefficient.

	SNR/dB	MSE/dB	NCC
Original Signal	−5.3024	0.9356	1
EMD	15.8726	0.4067	0.772
EEMD	21.6218	0.1068	0.985
CEEMDAN-ApEn	28.2298	0.0326	0.993

**Table 4:** Noise rejection ratio (NRR) comparison of de-noised PD signal.

	NRR/dB
EEMD	16.5825
CEEMDAN-ApEn	28.3012
